# Sleep and Rhythmic Profile After Pineal Gland Removal in Humans

**DOI:** 10.1111/jsr.70171

**Published:** 2025-08-13

**Authors:** Renata de Andrade Prado Gobetti, Clarissa Bueno, Letícia M. S. F. A. Soster, Anna Carolina de Campos de Barros Luvizotto Monazzi, Fernanda Gaspar do Amaral, Andréa Maria Capellano, Nasjla Saba da Silva, José Cipolla‐Neto

**Affiliations:** ^1^ Department of Neurology of the Clinical Hospital, Faculty of Medicine University of São Paulo São Paulo Brazil; ^2^ Children's Institute of the Clinical Hospital, Faculty of Medicine University of São Paulo São Paulo Brazil; ^3^ Department of Physiology Federal University of São Paulo São Paulo Brazil; ^4^ Division of Pediatric Oncology, Pediatric Oncology Institute/GRAACC Federal University of São Paulo São Paulo Brazil; ^5^ Department of Physiology and Biophysics, Institute of Biomedical Sciences University of São Paulo São Paulo Brazil

**Keywords:** actigraphy, circadian rhythm, melatonin, pineal gland tumour, pinealectomy, sleep disorders

## Abstract

Melatonin, a hormone produced by the pineal gland, is classically described as a central circadian modulator. However, the impact of its absence on circadian rhythmicity in humans remains poorly understood. Pinealectomised patients, in whom melatonin secretion is chronically suppressed, represent a valuable clinical model to investigate the physiological role of this hormone in sleep and temporal organisation. This study evaluated sleep quality and sleep disorders, as well as circadian rhythmicity, in 17 individuals who underwent surgical or radiotherapeutic treatment for pineal tumours and exhibited confirmed absence of circulating melatonin. Participants were assessed through structured clinical interviews, standardised sleep questionnaires, sleep diaries and actigraphy recording for a minimum of 15 consecutive days. Rhythmic analysis was performed for sleep/wakefulness and rest/activity data. Mild nocturnal sleep symptoms were reported, but diurnal complaints were frequent. Despite the chronic absence of melatonin, all subjects maintained a 24‐h sleep/wake cycle, and none met criteria for circadian sleep/wake disorders. We conclude that melatonin demonstrated not to be crucial for the maintenance of the circadian sleep/wake cycle in individuals with chronic melatonin absence due to pineal gland removal and minor sleep complaints are present in this population. Other synchroniser mechanisms possibly involving non‐photic entrainment might play an important role in the maintenance of the circadian rhythm.

## 1 Introduction

Melatonin produced by the pineal gland is recognised as a temporal inner marker and plays multiple physiological roles, including the circadian signalling for the sleep/wake cycle (do Amaral and Cipolla‐Neto 2018). Studies about melatonin physiology are mostly based on non‐human animal models, such as pinealectomised rodents. Transposing results about the activity/rest cycle of nocturnal animals to humans can be challenging, but studies in humans are limited for ethical issues.

Pineal gland tumours are a rare condition and represent 0.4%–1% of intracranial tumours (Rousselle et al. 2015). Pineal gland tumours are related to reduced levels of circulating melatonin, although a normal profile or even increased melatonin production is also reported (Slawik et al. 2016; Vorkapic et al. 1987; Mandera et al. 2011). Mandera et al. (2011) found undetectable melatonin levels in half of the studied patients. The tumour treatment involves surgical resection or radioablation of the gland, leading to the absence of circulating melatonin. Despite this, melatonin replacement after pineal gland removal is not a common clinical practice, and therefore pinealectomised patients due to pineal gland tumours are an interesting population to investigate melatonin function in humans.

Studies about the clinical consequences of disturbed melatonin profile in patients with pineal gland tumours are scarce and restricted to case reports or small series, focusing on investigating melatonin levels as a biomarker of the histological tumour type (Mandera et al. 2011; Barber et al. 1978; Leston et al. 2009). Some case reports suggest the association of low melatonin levels in patients with pineal gland tumours and sleep disturbances, such as severe insomnia, that could improve after melatonin replacement (Etzioni et al. 1996; Jan et al. 2001; Petterborg et al. 1991). Krieg et al. (2012) investigated sleep complaints in a series of nine patients, finding an increased insomnia proportion in pinealectomised patients compared to controls, but not to other patients undergoing craniotomy. Slawik et al. (2016) was the only polysomnographic study, finding no changes before and after the pineal gland removal. Studies addressing melatonin function related to circadian rhythmicity in this population are missing. Considering the main role of the pineal gland in the timekeeping system, it would be expected that these patients present sleep circadian rhythm disorders, as suggested by the few case reports (Barrera et al. 2023; Ferri et al. 2017; Májovský et al. 2017).

The aim of this study is to investigate the baseline sleep and circadian profile of patients with pineal gland tumours after surgical or radioablation treatment, in the context of absence of circulating melatonin. We hypothesised that patients with undetectable melatonin levels would report sleep disturbances and experience disruption of the circadian sleep/wake rhythm.

## 2 Methods

It was a cross‐sectional study conducted in three centres, the Clinical Hospital of the Faculty of Medicine of the University of São Paulo (FMUSP), the Institute of Biomedical Sciences of the University of São Paulo and the Support Group for Adolescents and Children with Cancer (GRAAC) of the Federal University of São Paulo. The study was approved by the Ethics Committee of the involved institutions, and patients and caregivers signed the informed consent, according to the Helsinki Declaration Statement.

Participants were enrolled at the Neurology Department and the Children's Institute of the Clinical Hospital, FMUSP and at GRAAC, Federal University of São Paulo.

Patients with treated pineal gland tumours at least 3 months after surgical gland resection or radioablation were enrolled, with no age limits. Eligibility criteria also included a confirmed histopathological diagnosis of the tumour, no recurring brain tumour after pineal ablation and subsequent chemotherapy, no visual loss and absence of circulating melatonin previously measured by urinary 6‐Sulfatoximelatonin (6‐SM) or plasmatic melatonin.

### 2.1 6‐Sulfatoximelatonin Measurement

Overnight urinary 6‐SM was collected and only patients with undetectable levels were included. The urine samples were collected from all subjects from 7:00 PM to 7:00 AM and kept in an opaque container. Samples were homogenised, had their volumes assessed and were kept under −80°C prior to the assay. 6‐SM levels were assessed using ELISA (IBL International, Hamburg, Germany), according to the manufacturer's instructions; results were expressed as nanograms of 6‐SM per milligram of creatinine.

### 2.2 Clinical Evaluation

A structured interview was conducted with all participants to collect demographic information, medical history and current or previous symptoms related to the tumour. This interview was always administered by either the first or second author of the study during the initial clinical visit, which included a neurological examination and the delivery of the actigraphy device. Medical records were reviewed to gather complementary data regarding diagnosis, histological type, tumour extension and treatment history. All participants underwent clinical, neurological and ophthalmological examinations on the same occasion.

The International Classification of Sleep Disorders (ICSD) 3rd edition criteria were used to define diagnosis for circadian sleep disorders, insomnia, parasomnia, narcolepsy, idiopathic hypersomnia, restless leg syndrome and sleep movement disorders. The presence or absence of sleep disorders was discussed among the first three authors when it was defined when there was total agreement. The Morningness–Eveningness Questionnaire (MEQ) developed by Horne Östberg and validated for the Brazilian population (Benedito‐Silva et al. 1990) was used to define chronotype, and the Munich Chronotype Questionnaire (MCTQ) (Shahid et al. 2011) was used to assess sleep mid phase and the difference between weekends and weekdays.

Patients were evaluated for sleep complaints using validated questionnaires. The Pittsburgh Sleep Quality Index (PSQI) (Buysse et al. 1989; Bertolazi et al. 2011) assesses sleep quality over the past month through seven domains, with global scores > 5 indicating poor sleep quality. The Epworth Sleepiness Scale (ESS) (Johns 1991; Bertolazi et al. 2009), including the child/adolescent version (ESS‐CHAD), evaluates daytime sleepiness in routine situations, with scores > 10 suggesting excessive sleepiness. The Insomnia Severity Index (ISI) (Morin et al. 2011) measures the severity and impact of insomnia symptoms over the preceding 15 days, with scores ≥ 15 indicating clinically significant insomnia. Fatigue was assessed by the Modified Fatigue Impact Scale (FI) (Fisk et al. 1994), which captures the impact of fatigue across physical, cognitive and psychosocial domains over the past month; scores above 38 are often interpreted as clinically relevant.

All questionnaires were self‐administered and completed by the patients themselves or by a legal guardian in the case of underage participants.

Sleep/wake pattern was assessed with sleep diaries filled continuously for a minimum of 15 consecutive days. Routine activities and medication intake were also registered in diaries. Average sleep onset and wake up times as well as sleep duration and the presence and number of naps were obtained from sleep diaries.

Activity/rest data were continuously recorded by actigraphy with ACT TRUST – Model: AT0503, Condor Instruments Ltda (São Paulo, SP, Brazil) attached to the left wrist for at least 15 consecutive days. Data were recorded in 1 min intervals.

### 2.3 Analysis

Demographic and clinical data are represented as proportions for categorical variables and median and interquartile range for continuous variables.

The sleep assessment tools raw values are presented as median and interquartile ranges. Normative population data were considered to define sleep disturbance as follows: PSQI value over 5 defined bad sleep quality; ESS over 10 defined excessive daytime sleepiness. ISI classifies insomnia as no insomnia symptoms (0–7), mild insomnia (8–14); moderate insomnia (15–21) and severe insomnia over 21.

Sleep/wake data collected with diaries were divided into 10 min blocks, and each epoch was categorised as sleep or wake for subsequent rhythmic analysis.

Actograms were constructed for visual inspection of the sleep/wake cycle pattern with data derived from sleep diaries and the activity/rest pattern derived from actigraphy. Sleep onset and sleep duration were defined by the combined evaluation of actigraphy and sleep diaries. The entire time series recorded by actigraphy for each subject was used for all rhythmic analysis.

Sokholove and Bushell periodograms were conducted to identify the period and the percentage of variance of possible rhythms present in sleep/wake and activity/rest cycles. This technique uses the Chi square distribution to detect rhythmicity in a time series (Oliveira et al. 2024) and periods from 2 to 28 h were screened. The percentage of variance represents the contribution of the identified period to the overall rhythmicity, and the significance threshold was set at 0.05.

Acrophase and the rhythm amplitude for a 24 h rhythm, if present, were assessed by the Cosinor method, which fits a cosine curve through the minimum square method. Non‐parametric rhythmic analysis (van Someren et al. 1996) was conducted to assess the 10 h of most activity (M10) and the 5 h with least activity (L5). The fragmentation of the 24‐h rhythm was assessed by the intradaily variability, based on successive time intervals, with values ranging from ≈0 for a perfect sine wave to ≈2 for Gaussian noise (Makarem et al. 2024; Mitchell et al. 2017). The relative amplitude is a summary measure of the robustness of the rhythm to assess the synchronisation with the average 24‐h cycle calculated from M10 and L5 counts and timing. Although definitive cut‐off values are not defined in the literature, higher RA values are indicative of more robust circadian rhythm and populational studies (Wallace et al. 2023) suggest that values above 0.8 would be expected for teenagers and adults. The rhythmic analysis was conducted with El Temps software, Barcelona, Spain (Diez‐Noguera 2025).

Correlation between time after pinealectomy and PSQI, ESS, ISI and FI scores as well as between age at diagnosis and the same variables was tested with Spearman correlation with the aim of investigating if the time elapsed without circulating melatonin or an earlier age of the procedure could be related to sleep symptoms.

A subgroup analysis was conducted comparing participants with tumours restricted to the pineal gland region and patients with tumours affecting the hypophysis gland as this last group has other metabolic abnormalities that could impact sleep. Wilcoxon test (*p* < 0.05 for significance) was performed comparing sleep onset, sleep duration, PSQI global score, ESS and ISI scores. All analyses were performed using STATA software (StataCorp. 2023. Stata Statistical Software: Release 18. College Station, TX: StataCorp LLC).

## 3 Results

Twenty‐one participants were selected for the study; clinical data were incomplete for one participant, and three withdrew after the initial assessment. Seventeen participants remained in the final sample. The median age was 16 (IQR: 12–20) and only four patients were female. Germ cell tumour was the most common histological type, and only four patients had lesions also affecting the hypophysis gland. The time elapsed since the pineal tumour resection or radioablation was 2 years or more for 15 patients (88%). Participants' data are summarised in Table 1.

**TABLE 1:** Demographic and clinical data.

Participant	Sex	Age (years)	BMI (kg/m^2^)	Tumour hystology	Age at diagnosis (years)	Time since pinealectomy (years)
1	M	16	27.4	GCT non‐germinomatous	8	7
2	M	15	24.9	GCT non‐germinomatous	5	5
3	M	8	16.8	GCT non‐germinomatous	6	2
4	M	20	21.7	GCT germinoma	10	10
5	M	15	16.6	GCT non‐germinomatous	10	4
6	F	17	25.3	Pineoblastoma	12	4
7	M	20	18.6	GCT germinoma	13	6
8	M	11	18.5	GCT non‐germinomatous	10	11 months
9	F	12	19.1	Pineoblastoma	6	5
10	F	11	14.2	Pineoblastoma	10	4 months
11	M	29	22.9	GCT germinoma	9	19
12	M	21	29.6	GCT germinoma	16	2
13	F	14	21.4	GCT germinoma	9	3
14	M	20	27.2	GCT germinoma	17	3
15	M	19	22.7	Pineoblastoma	12	7
16	M	16	19.4	GCT germinoma	9	7
17	M	15	18.1	GCT germinoma	13	2

Abbreviations: BMI, body mass index; GCT, germ cell tumour.

Sleep complaints were reported in the interview by half of the participants and included snoring (*n* = 4), restless sleep (*n* = 2), night awakenings (*n* = 2), daytime sleepiness (*n* = 4) and sleep walking (*n* = 1). Although not reported in the interview, another patient had night awakenings more than two times a week and diurnal naps of more than 1 h at least once a week, reported in the sleep log. Other frequent diurnal symptoms were attention impairment, anxiety, irritability and fatigue.

Median reported sleep duration was 8.4 h (IQR: 8–8.9). Overall sleep diaries were in agreement with actigraphy data in terms of the sleep onset and ending for the main sleep episode. All patients had inadequate sleep habits, and only one patient did not report sleep misalignment due to social constraints (social jet lag).

The neurological evaluation revealed visual field impairment (*n* = 3), strabismus (*n* = 6), cataract (*n* = 1), hearing impairment (*n* = 1) and motor deficit (*n* = 1).

Regarding sleep quality, the PSQI revealed scores ranging from 0 to 7. Only three participants scored above the clinical threshold for bad sleep quality (5–10), and none had scores suggestive of severe sleep impairment (> 10). ESS scores ranged from 5 to 16. While most participants scored within the normal range (0–10), diurnal excessive somnolence was reported by five. The ISI scores ranged from 0 to 17. Five patients had borderline scores for insomnia (8–14) and one scored as moderate insomnia evaluated by ISI. Finally, fatigue symptoms measured by the Modified Fatigue Impact Scale (FI) ranged from 11 to 70. Using the cut‐off of > 38 to indicate clinically meaningful fatigue, six participants were affected. Overall, 41% of the sample had diurnal complaints.

There was no significant correlation of time since pineal gland resection or ablation or age at diagnosis and PSQI, ISI, ESS and FI scores.

No patients filled criteria for circadian sleep disorders according to the ICSD (Table 2). According to chronotypes, 30% were classified as intermediate, 41.6% were morning chronotypes and 25% were evening chronotypes. The sleep mid phase ranged from 3 h 53 min to 4 h 45 min. Interestingly, the participant with the latest sleep mid phase had self‐identified as morning chronotype. Sleep mid phase ranged from 3 h 50 min to 4 h 10 min for the other participants identified as morning as well as intermediate chronotypes and ranged from 4 h 30 min to 4 h 40 min for the evening chronotypes.

**TABLE 2:** Number of individuals diagnosed with sleep disorders by clinical assessment.^a^

Sleep disorder	Number of diagnosed participants (total *N* = 17)
Insomnia	2
Parasomnia	1
Sleep related movement disorder	0
Sleep circadian rhythm disorder	0
Insufficient sleep	2
Snoring	4
Excessive diurnal somnolence	5
Bad sleep quality	3

^a^ Clinical assessment refers to a standardised set of questions, administered by a Sleep Medicine physician, with all patients receiving the same sequence of inquiries.

**FIGURE 1:**
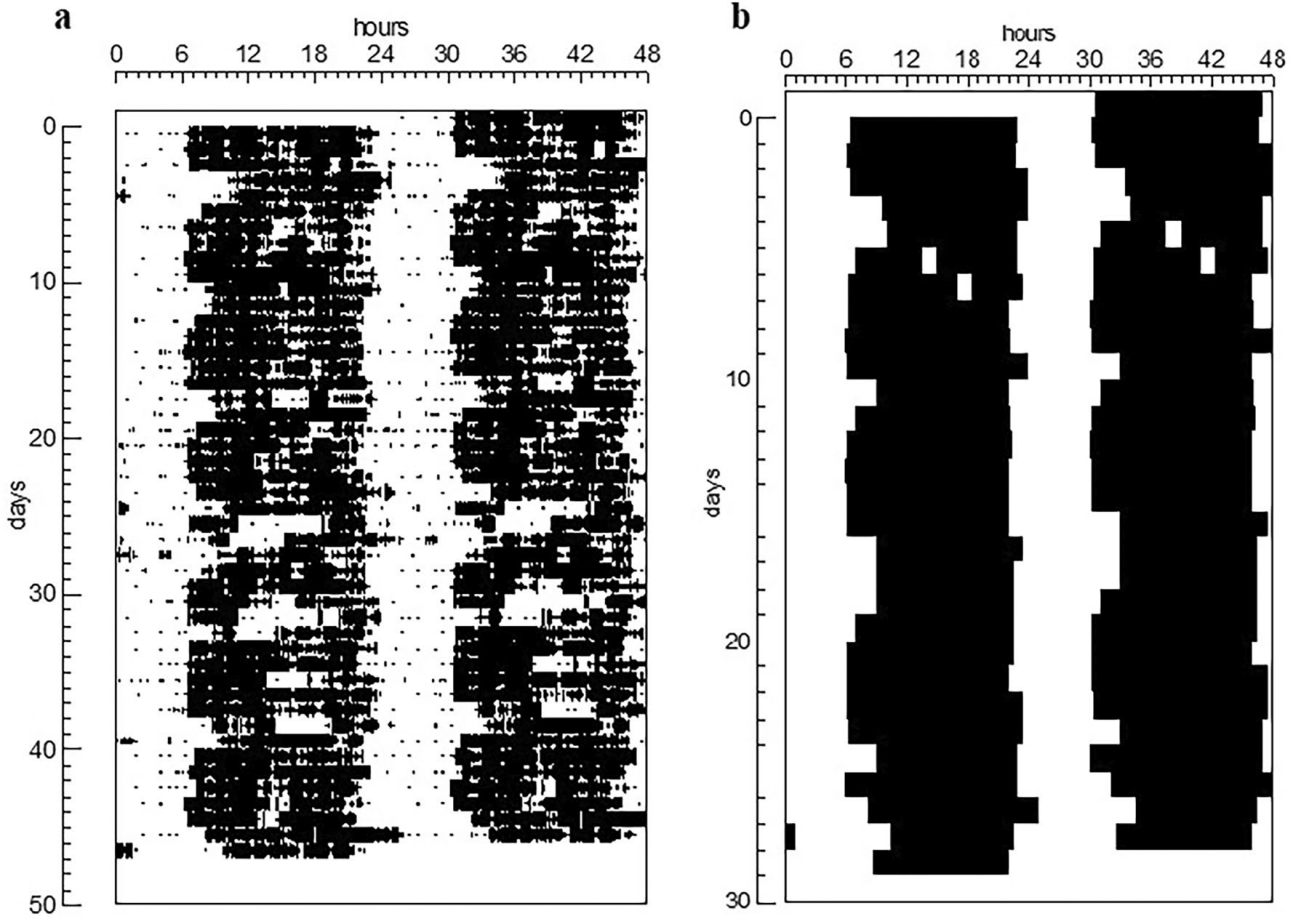
Actogram in double representation of participant 3: (a) represents actigraphy data and (b) represents sleep/wake pattern obtained from diaries for the same period. Black bars represent activity/wakefulness and white spaces represent rest/sleep.

Activity/rest data were recorded for a median of 28 days (IQR: 28–33) and revealed a clear circadian activity/rest rhythm for all subjects. Figure 1a presents the actogram for the activity/rest cycle and Figure 1b presents the actogram of sleep/wake data obtained from diaries for a representative participant (subject 3); this subject was chosen for having the longest time series recorded. The only significant periods identified in the periodogram analysis were the 24 h rhythm and the 12 h rhythm. Only three subjects showed relative amplitude (RA) values lower than 0.8, and 53% of our sample had RA over or equal to 0.9, confirming the robustness of the synchronised circadian rhythm. Overall, actigraphy data are summarised in Table 3, and detailed individual data are available in the Supporting Information.

**TABLE 3:** Actigraphy variables for the overall sample.

Days of actigraphy recording [median; IQR]	28 [26–33]
Sleep onset (mean; SE)	22 h 07 min (1 h 10 min)
Sleep duration (mean; SD)	8 h 24 min (54 min)
Activity acrophase (minimum and maximum)	11 h 54 min–17 h 25 min
Percentage of variance of the 24‐h rhythm by SB [median; IQR]	26.93 [23.65–31.19]
Intradaily variability (minimum and maximum)	0.47–0.98
Relative amplitude [median; IQR]	0.9 [0.82–0.93]

*Note*: Acrophase and the percentage of variance results refer to the 24‐h rhythm.

Abbreviation: SB, Sokolove‐Bushell periodogram.

The sleep logs analysis corroborated the actigraphic findings, also revealing a circadian sleep/wake pattern for all participants (Figure 1b); although five of them exhibited irregularity in sleep and wakefulness onset. Sokholove and Bushell periodograms identified the presence of a 24 h rhythm for all participants, and no other period was significant. Intradaily variability was lower than 0.1 for all participants, which aligns with the few reports of naps and night awakenings.

Comparing participants with tumours restricted to the pineal gland (*n* = 13) and patients also with hypophysis lesions (*n* = 4) we found no significant differences between groups for demographic data or for sleep parameters and scale scores. Only a marginal difference was found in age at diagnosis (*p* = 0.045). These results are summarised in Table 4.

**TABLE 4:** Sleep scales subgroup analysis.*

	Tumour restricted to pineal region (*N* = 13)	Tumour non‐restricted to pineal region (*N* = 4)	Overall	*p* **
Age	16 [12–19]	18 [13.5–21]	16 [12–20]	0.49
Age at diagnosis	10 [8–10]	11 [14.5–16.5]	10 [9–12]	0.045
Time since pinealectomy	5 [4–7]	2 [2.5–3]	4 [2–7]	0.08
ISI	6 [2–10]	5 [2.5–8]	6 [2–10]	0.73
ESS	7.5 [6–9.5]	7.5 [3–14.5]	7.5 [5.75–11]	0.95
PSQI	3 [2–5]	4 [1.5–5.5]	3 [2–5]	0.86

Abbreviations: ESS, Epworth Sleepiness Scale; ISI, Insomnia Severity Index; PSQI, Pittsburgh Sleep Quality Index.

* Median and interquartile range for the overall sample and each group.

** *p*‐value for comparisons between the group with tumour restricted to the pineal gland region and the group with combined hypophysis lesion.

## 4 Discussion

This study identified that individuals with chronic absence of circulating melatonin due to pineal gland tumour had minor sleep complaints and surprisingly had not developed sleep circadian rhythm disorder. The time elapsed since pineal gland resection or ablation had no influence on sleep parameters.

Melatonin is considered a temporal inner marker due to its timed synthesis regulated by the suprachiasmatic nuclei (NSQ) and restricted to the dark phase of the light/dark cycle (do Amaral and Cipolla‐Neto 2018). Several studies have demonstrated the potential of melatonin in synchronising endogenous rhythms and promoting phase shifts (Cruz‐Sanabria et al. 2023; Skene 2003; Comai and Gobbi 2024). Sleep/wake rhythm is also synchronised to melatonin rhythm with increasing sleep drive after dim light melatonin onset (Cruz‐Sanabria et al. 2023; Skene 2003). However, experimental studies also argue that melatonin would not be obligatory to the regulation of the sleep/wake cycle as pinealectomised rats preserve their sleep/wake rhythm (Fisher and Sugden 2010), as well as mouse strains naturally knockdown for melatonin (Roseboom et al. 1998).

Pinealectomised patients due to pineal gland tumours represent an interesting group to study the effects of melatonin absence, enabling the characterisation of a clinical syndrome of hypomelatoninemia, and contributing to better understanding its potential therapeutic uses.

Some case reports have suggested a correlation between low circulating melatonin levels in patients with pineal region tumours and sleep disorders, such as severe insomnia, with potential improvement upon melatonin replacement therapy (Rousselle et al. 2015; Vorkapic et al. 1987; Mandera et al. 2011; Barber et al. 1978; Leston et al. 2009; Etzioni et al. 1996; Jan et al. 2001; Petterborg et al. 1991; Krieg et al. 2012; Barrera et al. 2023; Ferri et al. 2017; Májovský et al. 2017). However, larger studies focusing on melatonin levels have primarily aimed to identify histological markers (Vorkapic et al. 1987; Mandera et al. 2011; Barber et al. 1978; Leston et al. 2009).

In pineal region tumours, cases of hypermelatoninemia (secretory tumours), hypomelatoninemia (destructive tumours), or normal melatonin profiles may occur. Germinomas, in particular, may secrete melatonin, which can either follow a physiological rhythmic pattern or become significantly diminished due to the undifferentiated or invasive nature of the tumour (Vorkapic et al. 1987). Leston et al. (2009) suggested a potential correlation between the daily melatonin profile and tumour histology. Notably, the circadian rhythm of melatonin was preserved in patients with pineocytomas, implying that melatonin production might be maintained in pineal parenchymal tumours with greater histological differentiation (Leston et al. 2009). Conversely, patients with germ cell tumours demonstrated reduced melatonin levels throughout the 24‐h period.

Most of our patients had germ cell tumours; the time elapsed since the pineal gland resection or radioablation was more than 2 years, and all had undetectable 6‐SM levels at the moment of the study. We can assume that this would be enough time to develop circadian rhythm disorders. Besides, they were in a stable phase of the disease and had no recurring tumour. So, our evaluation occurred in patients under chronic absence of circulating melatonin with no other confounding factors related to disease and treatment. Importantly, the absence of significant correlations between time since pinealectomy or age at diagnosis and the sleep questionnaire scores suggests that the presence or absence of sleep complaints does not vary substantially with the duration of melatonin absence, pointing to a time‐independent effect.

Although previous single cases and small series reported sleep disturbance in these patients, probably it was related to the over publication of symptomatic cases. Krieg et al. (2012) conducted a study using sleep quality and insomnia questionnaires in a series of nine patients. They identified a higher prevalence of insomnia among pinealectomised patients compared to controls, though not when compared to patients undergoing craniotomy without pinealectomy. This finding suggests that such complaints may be associated with craniotomy itself rather than the pinealectomy (Krieg et al. 2012). Furthermore, Slawik et al. (2016) also evaluated sleep quality, diurnal somnolence and chronotype in a series of eight patients 1–3 weeks before surgery and after the recovery of the acute phase of treatment and found no differences between the two groups. We highlight that most of their patients had normal levels of circulating melatonin prior to surgery (Slawik et al. 2016).

Our study identified that even after several years without circulating melatonin, pinealectomised humans maintain a robust circadian sleep/wake rhythm and mostly minor nocturnal sleep disturbances are reported, while diurnal complaints of sleepiness and fatigue are more frequent. Biological rhythms are entrained to the cyclic environment through the photic pathway projecting from the retina to the suprachiasmatic nuclei in the anterior hypothalamus (Mistlberger 1992). This temporal information is spread throughout the organism by regulating the timed synthesis of melatonin (do Amaral and Cipolla‐Neto 2018; Mistlberger 1992) but also neural projections to other hypothalamic regions may play a role in chronoregulation. Also, non‐photic entrainment following different pathways plays a major role in synchronising biological rhythms, as demonstrated for food availability and social interaction (Costa Petrillo et al. 2024; Tassino and Silva 2024). Our results suggest that synchronisation pathways independent of melatonin are sufficient to maintain the rhythmic profile of the sleep/wake cycle in humans.

This study has limitations such as the subjective assessment of sleep impairment that may not represent the overall frame. However, there was agreement in the results of actigraphy and sleep diaries according to the presence of a robust circadian rhythm. We did not have a control group for comparisons; however, as we identified a robust synchronised circadian rhythm for all participants and no rhythm disorder according to ICSD‐3 criteria, even though numeric differences could have been identified in relation to a control group, it would not change the main finding and possibly could not be considered clinically meaningful in the absence of complaints. Furthermore, the scales used were already validated for the population with defined cut‐off values.

The cross‐sectional design of the study prevented a longitudinal evaluation of melatonin profile and sleep/wake rhythm. Furthermore, most of our sample is composed of youngsters, and physiological changes occur in sleep regulation during life. The longitudinal follow‐up of these patients will be important to assess whether new symptoms or disorders may arise along with the age‐related physiological changes in both the circadian and homeostatic regulation of the sleep/wake cycle. Studies assessing sleep complaints and circadian rhythm before and after melatonin replacement in this population will also be important to better understand the impact of melatonin absence in humans and also related to other melatonin functions beyond sleep. Finally, tumours also affecting the hypophysis lead to other hormone and metabolic abnormalities that could contribute to sleep disturbances. However, the small proportion of these patients in our sample limited subgroup comparisons. The future inclusion of more patients with tumours non‐restricted to the pineal gland may provide new findings.

Melatonin demonstrated not to be crucial for the maintenance of the circadian sleep/wake cycle in humans in our study. Other synchroniser mechanisms possibly involving non‐photic entrainment are sufficient and might play a more important role than previously expected. Investigating temporal challenges in this population could provide further insights on the regulation of the timekeeping system. Sleep/wake cycle profile and sleep impairment should be investigated in individuals with pineal gland tumours even though most patients will not demonstrate major disturbances. Melatonin also plays other functions in the endocrine, cardiovascular and immune systems, as well as an antioxidant action. These functions should be considered beyond sleep to recommend melatonin replacement in patients with absence of this hormone.

## Author Contributions

**Renata de Andrade Prado Gobetti:** conceptualization, investigation, writing – original draft, methodology, validation, visualization, writing – review and editing, software, formal analysis, data curation. **Clarissa Bueno:** conceptualization, investigation, writing – review and editing, software, formal analysis, project administration, data curation, supervision, resources. **Letícia M. S. F. A. Soster:** conceptualization, investigation, writing – review and editing, software, formal analysis, project administration, data curation, supervision, resources. **Anna Carolina de Campos de Barros Luvizotto Monazzi:** data curation. **Fernanda Gaspar do Amaral:** conceptualization, investigation, data curation, funding acquisition, methodology, validation, project administration, supervision, resources. **Andréa Maria Capellano:** conceptualization, data curation, supervision. **Nasjla Saba da Silva:** conceptualization, data curation, supervision. **José Cipolla‐Neto:** conceptualization, investigation, funding acquisition, writing – review and editing, project administration, supervision, resources.

## Ethics Statement

The study was approved by the Ethics Committee of the involved institutions, and patients and caregivers signed the informed consent, according to the Helsinki Declaration Statement.

## Conflicts of Interest

The authors declare no conflicts of interest.

## Supporting information


**Table S1:** Raw actigraphy data of each patient.

## Data Availability

The data that support the findings of this study are available on request from the corresponding author. The data are not publicly available due to privacy or ethical restrictions.
