# Supplementary material for: Sleep and Rhythmic Profile After Pineal Gland Removal in Humans
Source: J Sleep Res. 2025 Aug 13;35(2):e70171. doi: 10.1111/jsr.70171 (PMC13003271; doi:10.1111/jsr.70171)
Supplement: Supplementary file 1 — Table S1: Raw actigraphy data of each patient. [file JSR-35-e70171-s001.docx]

Supplementary Material

**Table S1:** Raw actigraphy data of each patient

| **Participant** | **Number of days of actigraphy recorded** | **Main period (minutes)** | **Percentage of Variance (Sokolove & Bushell periodogram)** | **Acrophase (Cosinor)** | **Amplitude of the circadian rhythm (Cosinor)** | **Intradaily variability (IV)** | **Relative amplitude (RA)** | **Average sleep onset** | **Average sleep duration** |
| --- | --- | --- | --- | --- | --- | --- | --- | --- | --- |
| 1 | 24 | 1445 | 34,19 | 15h00min | 19600 | 0,616 | 0,9 | 0:53 | 7h54min |
| 2 | 15 | 1445 | 16,78 | 17h25min | 11319 | 0,554 | 0,64 | 22:01 | 9h30min |
| 3 | 49 | 1440 | 29,66 | 15h12min | 32837 | 0,471 | 0,94 | 22:58 | 8h05min |
| 4 | 28 | 1440 | 37,89 | 15h9min | 13577 | 0,584 | 0,93 | 23:34 | 7h35min |
| 5 | 26 | 1435 | 23,65 | 14h10min | 12094 | 0,723 | 0,82 | 22:49 | 6h46min |
| 6 | 29 | 1435 | 21,53 | 14h43min | 8211 | 0,654 | 0,9 | 23:17 | 8h46min |
| 7 | 29 | 1440 | 25,53 | 14h13min | 17673 | 0,689 | 0,95 | 23:05 | 8h26min |
| 8 | 23 | 1440 | 44,65 | 16h38min | 29228 | 0,576 | 0,92 | 0:07 | 8h13min |
| 9 | 26 | 1435 | 27,21 | 15h56min | 7749 | 0,684 | 0,93 | 23:25 | 9h15min |
| 10 | 27 | 1440 | 39,72 | 14h35min | 23656 | 0,479 | 0,95 | 22:26 | 7h56min |
| 11 | 28 | 1445 | 24,21 | 11h54min | 576 | 0,653 | 0,83 | 21:18 | 6h50min |
| 12 | 36 | 1440 | 10,13 | 15h18min | 1598 | 0,802 | 0,62 | 22:30 | 10h00min |
| 13 | 34 | 1440 | 15,04 | 15h49min | 15522 | 0,5 | 0,74 | 0:30 | 8h30min |
| 14 | 36 | 1445 | 28,39 | 14h25min | 11903 | 0,729 | 0,86 | 22:10 | 9h36min |
| 15 | 33 | 1445 | 24,39 | 13h40min | 9856 | 0,633 | 0,81 | 22:00 | 7h30min |
| 16 | 28 | 1440 | 31,19 | 13h18min | 19204 | 0,817 | 0,9 | 20:50 | 8h48min |
| 17 | 19 | 1440 | 26,93 | 13h6min | 8692 | 0,985 | 0,83 | 20:54 | 8h43min |
